# The effects of losartan on memory performance and leptin resistance induced by obesity and high-fat diet in adult male rats

**Published:** 2014-01

**Authors:** Seyydeh Gohar Sharieh Hosseini, Saeed Khatamsaz, Mehrdad Shariati

**Affiliations:** 1 Islamic Azad University, Kazerun Branch, Department of Physiology, Kazerun, Iran; 2 Islamic Azad University, Zarghan Branch, Iran

**Keywords:** Leptin resistance, Losartan, Learning, Memory performance, Morris water maze

## Abstract

***Objective(s):*** Leptin is a hormone secreted by adipose tissue and is involved not only in the regulation of feeding and energy expenditure, but also its role in memory enhancement has been demonstrated as well. The partial transfer of leptin across the blood-brain barrier in obese individuals causes leptin resistance and prevents leptin reaching brain. On the other hand, studies have shown that angiotensin antagonists such as losartan can improve memory and learning abilities. The aim of this study was to evaluate the effects of losartan on improving memory and leptin resistance induced by high fat diet in obese rats.

***Materials and Methods:*** 40 Wistar male rats were divided in 4 groups: control (C), losartan (LOS), high-fat diet (HFD) and high-fat diet and losartan (HFD and LOS). The spatial memory performances of the rats were assessed in the Morris water maze after 2 months of treatment. Then they were weighed and serum levels of leptin and triglyceride were measured.

***Results:*** In spite of receiving high-fat diet, no significant differences in body weight were observed in the (HFD & LOS) group. In the Morris water maze trial, the (LOS) and (HFD & LOS) groups also showed a significant reduction (*P* <0.05) in latency and path length. In addition, a significant decrease (*P* <0.05) in serum levels of leptin and no significant difference in serum levels of triglyceride was observed in the (HFD & LOS) group.

***Conclusion:*** Losartan can improve leptin resistance induced by obesity and high fat diet. At the same time, it modulates body weight and enhances learning and memory.

## Introduction

One of the main factors in human mortality, particularly in developed countries, is cardiovascular disorders which are often associated with diet and obesity. The close relationship between the factors involved in cardiovascular disorders on one hand, and learning and memory, on the other hand is well known (1, 2). One of these factors is the renin-angiotensin system (RAS) (3, 4). Studies have shown that angiotensin II can affect neurons, particularly in brain regions involved in memory and cognition (such as the hippocampus and amygdala), and leave behind destructive and neurodegenerative effects, leading to disruptions in cognitive and memory performances (3, 4).

Angiotensin antagonists, which are used as antihypertensive drugs are divided into two categories: 1. Angiotensin Converting Enzyme-Inhibitor or ACE-Inhibitor, such as captopril, perindopril etc, and 2. angiotensin-receptor blockers, such as losartan, valsartan etc. Angiotensin receptors (AT1 and AT2) have been observed in many regions of the brain, particularly in areas related to learning and memory functions (e.g., hippocampus, amygdala, thalamus, sabstantia nigra, locus coeruleus and prefrontal lobes) (5, 6). Therefore, angiotensin antagonists can improve brain disorders and enhance memory and learning abilities (7, 8). In contrast, it has been reported that centrally administered Ang II improves aversive memory (9), but using similar learning tasks, others have shown that this peptide has no action at all on memory retention (10, 11).

Moreover, the leptin receptors are widely distributed in the regions of the brain such as the hippocampus, thalamus, prefrontal cortex, cerebellum, midbrain, brain stem and neocortex (12). The 16 kDa protein leptin is released from adipose tissues into the blood stream and mediates a variety of central and peripheral functions through its receptors (13, 14). This peptide reduces the clearance of the neurotrophic factor from hippocampal neurons (15), and is involved in the formation of hypothalamus in the early stages of development and can elevate cognition (16). It has been shown that direct administration of leptin into rat brain improves memory processing and increases N-methyl-D-aspartate (NMDA) receptors (17). Other study reported that leptin receptor-deficient obese rodents showed the Impairment of long-term potentiation and spatial memory. Furthermore they showed impaired learning in the water maze in comparison with normal rodents (18).

Leptin plays a key role in regulating energy intake and energy expenditure, including appetite, hunger and metabolism and controls body weight (19). This hormone is passed through the blood-brain barrier into the central nervous system (CNS) and suppresses appetite by suppressing the production of neuropeptide Y that plays an important role in the regulation of appetite and body weight (20, 21). In addition, the Leptin stimulates the sympathetic processes in the CNS. Sympathetic terminals entering into the peripheral tissues, such as adipose, through the effect of epinephrine and norepinephrine neurotransmitters, produce cAMP in the cytosol. The increase in the amount of cAMP suppresses production of leptin and activates lipase enzyme, which increases lipolysis. Thus, the response of CNS to the serum leptin level via a feedback path reduces adipose tissues leading to the inhibition of leptin production (22).

In obese individuals who are on high-fat diet, leptin feedback mechanism is disrupted, and they have a high leptin plasma level due to elevated levels of lipids (such as triglycerides), the partial transfer of leptin through blood-brain barrier and interference in the signaling of its receptors (23, 24). Therefore, obese people have a leptin resistance (25, 26). Moreover, as adipose tissues increase in obese individuals, expression of alpha 2-adernergic receptors on the surface of these tissues is increased and responses of beta-adrenergic receptors (as the main receptors of these tissues) disappear. This increase in alpha 2-adrenergic receptors, decreases cAMP production and lowers lipolysis in adipose tissues by inhibiting adenylyl cyclase system. Hence, the negative feedback mechanism which controls adipose tissues through sympathoadrenal system becomes an impaired cycle in obese people (27, 28).

Finally, the wide spread presence of RAS and leptin receptors in different parts of the central nervous system suggests that these systems are involved in development and activities of different parts of the brain. Several studies have shown close relationship between RAS and Alzheimer's disease, stroke memory, and learning alcoholism stress depression (29-31). Since its well demonstrated that angiotensin antagonists, such as losartan can be effective in learning and memory processes, in the present study we want to clarify the effects of losartan on leptin resistance and memory impairment caused by leptin resistance.

## Materials and Methods

***Animals***

Forty Wistar male rats weighing 270±20 g were provided by the Iranian Razi Institute and were housed in standard cages with free access to food and water. In order to adapt to new environmental condition all animals were kept in the Animal House of Kazerun Islamic Azad University for one week before entering into the trial. The animal house temperature was maintained at 23±3 °^C^ with a 12 hr light/dark cycle (light on from 06:00 to 18:00 hr). The ethical guidelines for the investigation of experimental animals were followed in all tests. All efforts were made to minimize animal suffering and to reduce the number of used animals.

***Treatments*** ***and drugs***

Animals were randomly divided into 4 groups of 10 including control (C) (left untreated), losartan (LOS) (receiving 20 mg/kg bw losartan plus standard diet), high-fat diet (HFD) (receiving only high-fat diet) and high-fat diet and losartan (HFD & LOS) group (receiving 20 mg/kg bw losartan plus high-fat diet). Losartan was dissolved in distilled water and orally administered to groups receiving losartan at 11 am every day for two months.

Two different diets were used in this experiment: (a) standard laboratory rodent’s chow made on the basis of NRC 1995 (32) by Pars Company for groups receiving standard diet, and (b) cafeteria diet for groups receiving high-fat diet. Cafeteria diet was composed of 15% protein, 20% - 50% carbohydrates and 20% - 40% fat (33). Cafeteria diets used in this experiment were of 4 types: (a) condensed milk and bread, (b), chocolate, biscuit and coconut powder, (c) boiled potatoes and cheese, and (d) popcorn. These four diets were consumed in a 4 days row by two groups (HFD and HFD & LOS) and were repeated in turn for 60 days (34).

After 2 months, animals were tested in the Morris water maze and latency and path length to find the hidden platform were recorded. Moreover, animals were weighed, blood samples were collected and serum levels of leptin and triglyceride were measured using m/r-ELISA kits (Made by Mediagnost Germany).

***Morris water maze***

The Morris water maze (MWM) is a test of spatial learning for rodents that relies on distal cues to navigate from start locations around the perimeter of an open swimming pool to locate a submerged escape platform. This maze was a circular pool, 120 cm in diameter and 60 cm in depth and filled with 20-22°C water 30 cm in depth. The pool was divided into four equal quadrants (North, East, West and South) and a transparent lucid platform (10×10 cm) was submerged 2 cm beneath the surface of the water, in the north-west quadrant of the pool. A video camera mounted at the height of 180 cm above the center of the maze and data was stored in a computer system. Maze was placed in a room with various prominent visual cues around it which remained in fixed positions throughout the experiment (35). Rats were trialed in Morris water maze. Each animal participated in five trials per day for five days. Each animal was given 60 sec to reach to the platform, upon which it remained for 10s. If the platform was not located within 60 sec the animal was placed on it by the experimenter. The next trial started immediately after removal from the platform.

**Figure 1:**
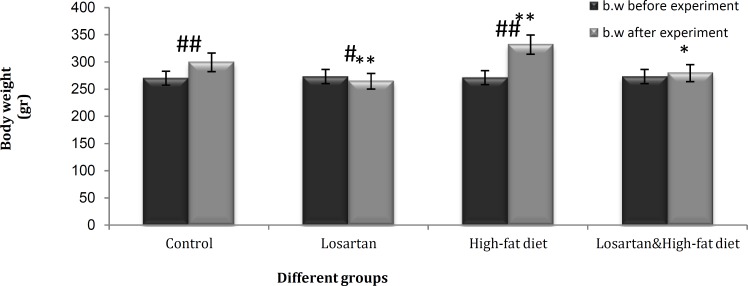
Mean body weights in different groups before and after the experiment. Statistical analysis was performed using ANOVA followed by Tukey's test for multiple comparisons for body weights after the experiment between groups and Paired t-test for before and after the experiment within a group. Data are shown as mean±SD. *P*<0.05 was the critical criterion for statistical significance. *P< 0.01, **P< 0.001 *vs* control after the experiment. #* P*<0.01, ##* P*<0.001 within a group before and after the experiment

After completion of the five trials, the animal was placed in its home cage. Escape latency and path length to find the hidden platform were recorded.

***Data analysis***

All data was expressed as mean±SD. Values for leptin and triglyceride serum concentration were compared by one-way ANOVA and followed by Tukey's test for multiple comparisons. Spatial task measures (latency and path length) were averaged within a group for five trials per day and analyzed using a repeated- measures ANOVA and followed by Tukey's test for multiple comparisons. The mean of body weight within a group before and after the experiment were compared using a paired t-test. All the statistical analysis was performed using SPSS 11.5 for Windows. *P<0.05 *was the critical criterion for statistical significance.

**Figure 2:**
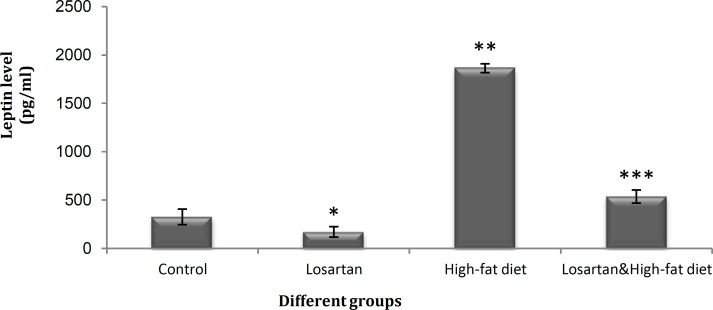
Serum leptin levels (pg / ml) in different groups. Statistical analysis was performed using ANOVA followed by Tukey's test for multiple comparisons. Data are shown as mean±SD. *P*<0.05 was the critical criterion for statistical significance. *P< 0.001, **P< 0.01 ***P< 0.05 *vs* control

**Figure 3:**
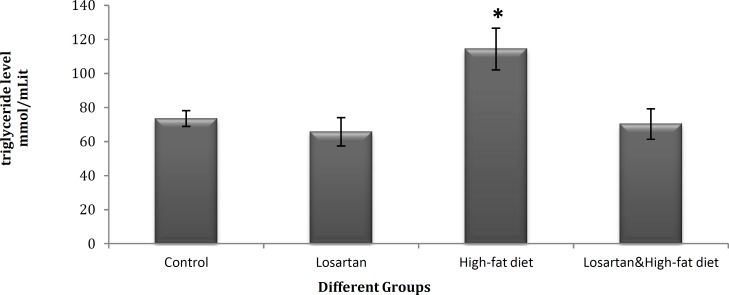
Serum triglyceride levels in different groups (mmol/ml). Statistical analysis was performed using ANOVA followed by Tukey's test for multiple comparisons. Data are shown as mean±SD. *P*<0.05 was the critical criterion for statistical significance. *P< 0.05 *vs* control

## Results

***The effects of losartan on body weight***

The mean body weights of different groups before and after the experiment were shown in Figure 1. The mean of body weight after the experiment between control and other groups were compared. A significant decrease in mean body weight was observed in the LOS (*P*<0.001) and HFD & LOS groups (*P*<0.01) and a significant increase was observed in HFD group (*P*<0.001) in comparison with the control group. 

The mean of body weight within a group before and after the experiment also were compared. The control and HFD (*P*<0.001) groups showed a significant increase in body weight and the LOS group (*P*<0.01) showed a significant decrease. The mean body weight in HFD & LOS group didn’t show significant difference even by receiving high-fat diet during the experiment (Figure 1). 

***The effects of losartan on serum leptin and triglyceride levels***

There was a significant difference in the serum level of leptin among the groups receiving the losartan (*P* <0.01), however, both were significantly lower than the level in HFD group (*P*<0.05) (Figure 2). Also, in comparison with control, the serum levels of leptin were increased in HFD (*P* <0.01) and HFD & LOS group (*P*<0.05) and reduced in LOS group (*P*<0.001) (Figure 2). Furthermore, no significant differences were observed in serum levels of triglyceride in HFD & LOS and LOS groups in comparison with control (*P*>0.05) (Figure 3). In respect to other groups, only HFD (on high-fat diet, without losartan) showed a significant increase (*P* <0.05) in serum triglyceride level (Figure 3).

**Figure 4:**
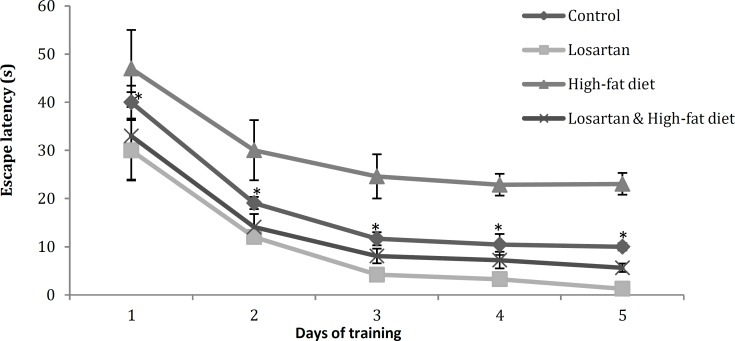
Escape latency (s) in the training days in different groups. Statistical analysis was performed using repeated-measures ANOVA. Data are shown as mean±SD. *P*<0.05 was the critical criterion for statistical significance. *P< 0.05 *vs* other groups

***Evaluation of escape latency and path length during training days*** ***in Morris water maze***

**Figure 5:**
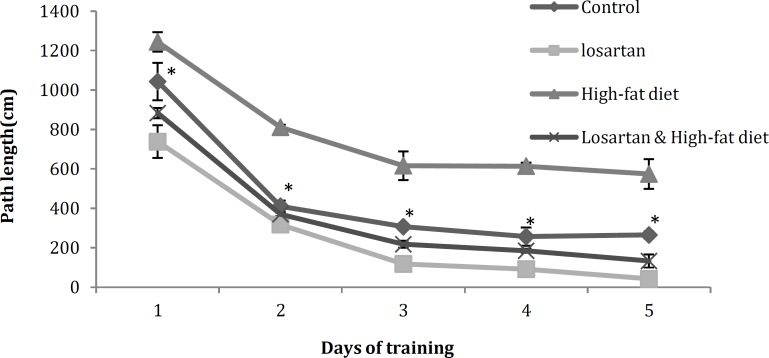
Path length (cm) in the training days in different groups. Statistical analysis was performed using repeated-measures ANOVA. Data are shown as mean±SD. *P<0.05 *was the critical criterion for statistical significance. *P< 0.05 *vs* other groups

Results of Morris water maze that were the latency and path length of finding the hidden platform during the five days of trial among various groups, were compared in Figure 4 and 5. Data were shown as mean (±) standard deviation of total latency and path length (for finding the hidden platform) of five trials for each group per day. As it has been shown in Figure 4 and 5, there were significant differences in escape latency and path length for finding the hidden platform between four groups in all of the five training days (*P*<0.05). 

Animals of HFD spent significantly more time and traversed longer distance to find the platform in the water maze (*P*<0.05). In contrast, although there is a significant difference between the groups receiving losartan (*P*<0.001), both showed a decrease in latency (*P*<0.05) and path length (*P*<0.05) in respect to Control and HFD. The differences among the training days in all of groups were statistically significant (*P*<0.05) (Figure 4, 5).

## Discussion

The present study demonstrated that losartan could improve leptin resistance even when cases were on a high-fat diet. As seen in Figure 2, losartan reduced leptin concentration in both LOS and HFD & LOS group. Leptin is released from adipose tissues into the blood stream and regulates appetite, feeding and energy expenditure (13, 14). Leptin can enter the central nervous system and inhibit the production of neuropeptide Y, which suppresses appetite and regulates the body weight (20, 21). A negative feedback mechanism controls fat and leptin levels through the sympathoadrenal system (22, 36). The partial transfer of leptin across the blood-brain barrier in obese individuals causes leptin resistance and prevents leptin to reach brain. The development of leptin resistance have several reasons: (a) interference in leptin transport due to an increase in lipids concentration such as triglycerides (22, 23), (b) partial transfer of leptin through the blood-brain barrier (23, 24), and (c) disruption of leptin receptor signaling (23). 

Leptin concentration in the HFD & LOS and LOS group were much lower than HFD (Figure 2). And serum triglyceride concentrations in these groups were very low compared to the HFD (Figure 3). Serum level of triglyceride is considered as a good indicator of lipolysis in adipose tissues. The increase of adipose tissue in obese individuals can cause elevation of the alpha 2-adrenergic receptors expression and disappearance of beta receptors responses. Alpha 2-adrenergic receptors reduce cAMP production and lipolysis in adipose tissue through the inhibition of adenylyl cyclase system. In this way, the negative feedback mechanism that controls fat and leptin levels through the sympathoadrenal system becomes a defective cycle in obese individuals (27, 28). Angiotensin II facilitates sympathetic processes and increases the release of noradrenaline from peripheral Sympathetic nerve terminals as well as catecholamines from adrenal medulla (22, 37, 38). Losartan may compensate the enhanced sensitivity of adrenergic system in obese patients by inhibition of AT1 receptors and reduction of alpha 2-adrenergic sympathetic activity. Therefore, losartan decreases alpha 2-receptors sensitivity and promotes beta receptors response that increases cAMP level and inhibits leptin synthesis. Also, interference in leptin transport across the blood-brain barrier by raising triglycerides (37, 38), is compensated by an increase in lipolysis and decline in serum triglycerides. On the other hand, losartan inhibits the effect of angiotensin II on endogenous growth factors secretion (7, 15), which increase liposynthesis through the insulin signal (23) and reduces the volume of adipose tissues. The weight loss and decreasing concentration of triglyceride in groups which received losartan (figure 1 and 3) could be an indication of improved leptin resistance, regulation of feeding and enhancement of lipolysis in adipose tissues.

The results of our experiment also have demonstrated that losartan can improve memory impairment caused by leptin resistance (Figure 4 and 5). The protective activities of leptin on nerve cells in the branching regions of brain involved in neurodegenerative processes (39, 40) have been demonstrated. This hormone reduces apoptosis in neuroblastoma cells (41) and has a protective effect on dopaminergic cells (42). It has been shown that direct administration of leptin into the hippocampus improves learning and memory performance (43, 44) and facilitates hippocampal long term potentiation (LTP) (45). Furthermore, leptin facilitates the conversion of short-term potentiation (STP) into LTP (46) and enhances LTP at hippocampal CA1 synapses (44). The synaptic activation of NMDA receptors and a concomitant postsynaptic rise in intracellular Ca2+ are pre-requisites for the LTP induction in the CA1 region (47). There is evidence that shows leptin evokes a novel form of NMDA receptor-dependent long-term depression (LTD) under conditions of enhanced excitability (48). Other study reported that leptin regulates neuronal excitability and cognitive function. Leptin enhances hippocampal synaptic plasticity and improves performance of rodents in learning and memory tasks (49).

Because of the interference in leptin functions and leptin resistance, this hormone cannot exert its beneficial effects on nerve cells in obese subjects on high-fat diet and the risk of cognitive disorders, such as Alzheimer's may increase in these people. Likewise, learning and memory performances in obese individuals are lowered in respect to their lean counterparts (50, 51).

As it has been illustrated in Figure 4 and 5, the LOS and HFD & LOS groups that have low concentration of serum leptin in comparison with HFD group, show significant decreasing in escape latency and path length. Thus the results of present experiment demonstrated that losartan improves leptin resistance and helps to transport of leptin across the blood-brain barrier and exert its beneficial effects on nerve cells and enhance learning and memory performance.

Several studies have reported that low doses of losartan, captopril, and perindopril can improve the disorders caused by scopolamine (52, 53). In another study, the beneficial effects of angiotensin II antagonists, such as losartan and Valsartan, on reduction of transfer latency caused by scopolamine have been shown (54). Similarly, the weakening and destructive effects of angiotensin II (7, 8) as well as Improving and enhancing effects of AT1 and AT2 antagonists on learning and memory processes have been well characterized (40, 55). In the present study, escape latency and path length to find hidden platform in water maze is reduced significantly in rats receiving losartan (Figure 4 and 5) which is considered as an indication of learning and memory strength. In the presence of losartan and other blockers, angiotensin II remains in circulation and is converted to angiotensin III and IV by blood aminopeptidases. Angiotensin IV increases the intracellular calcium level (not-associated with NMDA receptors) and strengthens memory (56). The presence of a large number of AT4 receptors in neocortex, hippocampus, amygdala and basal glands, involved in modulating learning, memory and cognitive processes, can confirm this issue (57).

Release of the neurotransmitter, acetylcholine is well established as playing an important role in memory processing. Therefore based on the high levels of Ang IV binding sites in regions containing cholinergic bodies or their terminal fields in conjunction with the facilitory effect of Ang IV on memory, the effect of Ang IV on acetylcholine release from hippocampal slices was investigated. Although Ang IV did not increase basal levels of acetylcholine release, it did increase depolarised induced release of acetylcholine from the hippocampus in a dose dependent manner (58). This suggests that potentiation of cholinergic transmission is one of the mechanisms by which Ang IV facilitates memory. In addition Ang IV has also been demonstrated to enhance LTP, a cellular basis for learning and memory (59).

## Conclusion

In summary losartan can lower leptin resistance and improve leptin transport across the blood-brain barrier by increasing lipolysis and reestablishment of leptin feedback mechanism. At the same time, it can reduce body weight through lowering triglyceride level and adipose tissues. In addition to its effects through conversion of angiotensin II to IV and other mechanisms that have been shown in previous studies, losartan can improve and enhance memory and learning performances by improving leptin resistance and facilitating leptin beneficial actions on CNS and memory enhancement.
